# Current understanding of the contribution of lactate to the cardiovascular system and its therapeutic relevance

**DOI:** 10.3389/fendo.2023.1205442

**Published:** 2023-06-15

**Authors:** Panyun Wu, Tengteng Zhu, Yiyuan Huang, Zhenfei Fang, Fei Luo

**Affiliations:** ^1^ Department of Cardiovascular Medicine, The Second Xiangya Hospital, Central South University, Changsha, Hunan, China; ^2^ Research Institute of Blood Lipid and Atherosclerosis, the Second Xiangya Hospital, Central South University, Changsha, China

**Keywords:** lactate, pathophysiology, cardiovascular disease, angiogenesis, signal transduction

## Abstract

Research during the past decades has yielded numerous insights into the presence and function of lactate in the body. Lactate is primarily produced *via* glycolysis and plays special roles in the regulation of tissues and organs, particularly in the cardiovascular system. In addition to being a net consumer of lactate, the heart is also the organ in the body with the greatest lactate consumption. Furthermore, lactate maintains cardiovascular homeostasis through energy supply and signal regulation under physiological conditions. Lactate also affects the occurrence, development, and prognosis of various cardiovascular diseases. We will highlight how lactate regulates the cardiovascular system under physiological and pathological conditions based on evidence from recent studies. We aim to provide a better understanding of the relationship between lactate and cardiovascular health and provide new ideas for preventing and treating cardiovascular diseases. Additionally, we will summarize current developments in treatments targeting lactate metabolism, transport, and signaling, including their role in cardiovascular diseases.

## Introduction

Lactate is an important metabolite mainly generated through glycolysis (1–3). Conventional wisdom holds the opinion that lactate is a metabolic waste produced in normal cells only under hypoxic conditions, including intense exercise and ischemia (1–3). However, burgeoning evidence points out that lactate is not an innocuous bystander metabolite as traditionally viewed, but instead serves as a fuel source for the myocardium (4). In addition, lactate plays special roles in regulating vascular smooth muscle cells (VSMCs) (5), promoting angiogenesis (6, 7), regulating hemodynamics (8), and cardiac electrophysiological activity (9, 10), all of which are essential for the maintenance of cardiovascular homeostasis. In addition, the finding of histone lactoylation (11) and non-histone lactoylation (12) is attractive. In light of the recent finding that lactoyl-CoA may be found in the cardiac tissue of mice (13), it would be prudent to investigate its influence on heart biology.

For over 90 years, it has been observed that lactate can be produced in functioning mitochondria that have sufficient oxygen. This observation is referred to as the Warburg effect (1–3). Studies have recently reported that the Warburg effect occurs in various pathophysiological states of the cardiovascular system, including atrial fibrillation, pulmonary hypertension (PAH), and heart failure (HF), suggesting it has critical roles in cardiovascular disease (14–18). Furthermore, numerous clinical and preclinical studies have shown that lactate directly affects various cardiovascular diseases.

Targeting lactate metabolism has led to a breakthrough in treating some diseases, especially in the field of cancer (19). Some recent studies have indicated that targeting the metabolism, transport, and signaling of lactate may be a promising method for preventing and treating cardiovascular diseases (20). In this review, we summarize how lactate regulates the cardiovascular system, highlighting how it affects cardiovascular diseases. Furthermore, we attempt to provide readers with a systematic and objective understanding of the effect of lactate on cardiovascular health, proposing new ideas about the pathogenesis of cardiovascular diseases and their treatment by targeting lactate.

## Lactate metabolism, transportation, and signal transduction

### Lactate production

**Figure 1:**
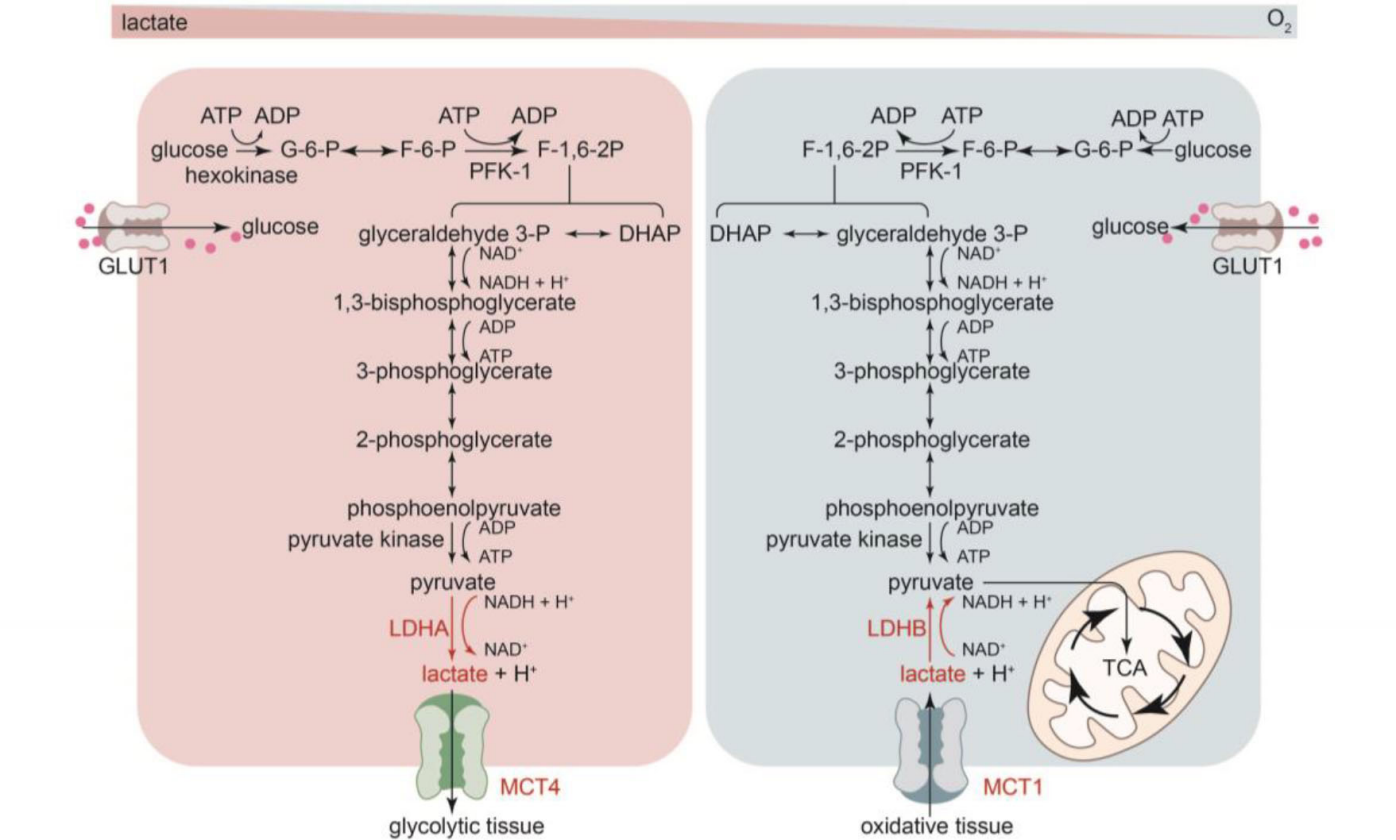
Metabolism and transport of lactate under conditions of varying oxygen availability. *DHAP* dihydroxyacetone phosphate, *F-6-P* fructose-6-phosphate, *F-1,6-2P* fructose-1,6-bisphosphate, *GAPDH* glyceraldehyde-3-phosphate dehydrogenase, *G-6-P* glucose-6-phosphate, *MCT* monocarboxylate transporters, *PFK-1* phosphofructokinase-1, *TCA* tricarboxylic acid cycle.

Lactate is mainly generated through glycolysis in most tissues of the human body, with the highest level of production detected in muscles (1–3). Under anaerobic conditions, pyruvate is reduced to lactate with nicotinamide adenine dinucleotide (NADH) and is subsequently fed into the Cori cycle as a substrate for gluconeogenesis. Glycolysis yields only two adenosine triphosphate (ATP) molecules and two lactate molecules per glucose without consuming any oxygen. Under aerobic conditions, pyruvate enters the Krebs cycle, producing abundant usable energy (approximately 25 ATP molecules per glucose) without lactate production (**Figure 1**).

### Lactate dehydrogenase

LDH mediates the bidirectional conversion of pyruvate and lactate and plays a crucial role in lactate metabolism (**Figure 1**) (1, 3, 19). LDH has two major isoforms—namely, LDHA and LDHB. LDHA predominantly catalyzes pyruvate reduction to lactate and couples NAD+ regeneration (21), whereas LDHB mostly converts lactate into pyruvate and couples NADH formation (21). Different combinations of LDHA and LDHB can entirely assemble into five tetrameric isozymes (LDH1, LDH2, LDH3, LDH4, and LDH5), which differ in their Km values for lactate and pyruvate, electrophoretic mobility, and sensitivity to pyruvate accumulation (22). Additionally, they differ in tissue expression; for instance, LDHA is the predominant isoform in skeletal muscles and other highly glycolytic tissues, whereas LDHB is the predominant isoform in the myocardium (21).

### Lactate transportation

Monocarboxylate transporters (MCTs) can bidirectionally transport protons and monocarboxylate ions (lactate, pyruvate, and ketone body molecules), depending on the concentration of both, protons and monocarboxylate ions, in the environment and/or cellular context (23, 24).The MCTs belong to the SLC16 gene family, and different MCT subtypes synergistically maintain the lactate balance. In normal tissues, MCT1 is mainly responsible for lactate transmembrane transport and plays a role both in the import and export of lactate, which depends on the transmembrane gradient for lactic acid (24, 25). In cells that generate high lactic acid concentrations, MCT4 is primarily responsible for facilitating lactic acid and H+ export across cell membranes (24). MCT2 is very similar to MCT1, whereas MCT3 is functionally similar to MCT4 (3). MCT-mediated transmembrane transport is the foundation of the “lactate shuttle” (19, 26) which describes lactate’s function in the transmission of oxidative and gluconeogenic substrates and in cell signaling (19, 26).

### Lactate signal transduction

Lactate can act as an extracellular ligand to conduct signals *via* G protein-coupled receptor 81 (GPR81). GPR81 is expressed in meningeal fibroblasts and adipocytes, where it inhibits lipolysis by decreasing the concentration of cyclic adenosine monophosphate e (27) and induces brain vascularization through ERK1/2 and Akt signaling (28). GPR81 also expresses in some tumor cells and sustains tumor growth and metastasis *via* triggering lactate-sensitive machinery (29). In recent years, researchers have determined that Gpr132, a member of the pH-sensing G protein-coupled receptor family, is a supplemental sensor/receptor for lactate and is highly expressed in macrophages (29). The lactate–Gpr132 axis stimulates the tumor–macrophage interplay to sustain breast cancer metastasis; nevertheless, its molecular mechanism requires further studies (29).

## Effects of lactate on the cardiovascular system

As the principal metabolite of glycolysis, lactate is a substrate for gluconeogenesis and energy metabolism and a signal molecule that regulates gene expression (11, 13, 30) and immune inflammation (11, 31–33) and promotes tumor growth (33–35). With an in-depth understanding of lactate’s regulatory function in tissues and organs, its effects on the cardiovascular system have gradually been revealed. Lactate can serve as energy source for the myocardium, regulate cardiac electrophysiological activity, modulate the function of VSMCs, and promote angiogenesis.

### Energy source for the myocardium

Under normal conditions, fatty acids are the main energy source for the heart (4, 36). However, when the heart is stressed by β-adrenergic stimulation (37–39), chronotropic challenge (40), increasing afterload (38, 41), or shock (42), lactate becomes the preferred fuel. The myocardium obtains 60–90% of its oxidizable carbon source from lactate (43–45). It is now generally accepted that dietary glucose is metabolized into lactate, which is then transported throughout the body and used to fuel the TCA cycle in tissues like the heart (46). It has been shown that the heart is the largest lactate-consuming organ in the body (47, 48). Almost all of the lactate absorbed by the myocardium is oxidized as fuel (39). Selective utilization of the fuel substrate is protective for the myocardium while meeting the energy demand (4). Interestingly, both embryonic and induced pluripotent stem cells can be differentiated into purified cardiomyocytes simply by growing them in glucose-free media containing lactate (49). Nevertheless, the diabetic myocardium constitutes an exception; in particular, compared to the control rat heart, the diabetic rat heart exhibits remarkably reduced lactate oxidation, which may cause an increase in the cytosolic NADH/NAD ratio (50). This observation indicates that diabetes causes specific inhibition of myocardial lactate oxidation, which may explain why patients with heart disease complicated by diabetes have a worse prognosis (50).

### Lactate regulates VSMCs

VSMCs, the main cellular components of the vasculature, are very important for the maintenance of vascular tension and the regulation of blood pressure (51–54). Recent studies have revealed that lactate promotes the proliferation (55), migration (56), and phenotype conversion of VSMCs (5). In an *in vitro* study, Kovacs et al. (57) observed that the promotion of lactate production resulted in a considerable increase in calpain activation in the pulmonary arterial smooth muscle cells (PASMCs) of patients with pulmonary arterial hypertension (PAH). Calpain inhibition prevents lactate-induced cell proliferation and reduces apoptosis. Furthermore, previous studies confirmed that the prevention of extracellular and intracellular lactate generation *via* downregulation of siRNA-PKM2 or LDHA inhibits the proliferation of human aortic VSMCs (16, 58).

The mobility of activated VSMCs is closely related to enhanced aerobic glycolysis (56, 58). Previous studies have shown for VSMC that the inhibition of glycolytic activity inhibits lactate production and migratory behavior *via* a compromised STAT3/HK2 signaling axis (56, 58). *In vitro* experiments conducted by Yang et al. revealed for the first time that lactate promotes the expression of synthetic VSMC markers instead of contractile markers when compared to lactate-free circumstances (5). Recent studies have also reported that lactate can inhibit arterial SMC contraction *via* Ca^2+^-activated K^+^ channels (K_Ca_ channels) (59, 60). Lactate regulates VSMCs through these pathways, suggesting a potential regulatory mechanism for vascular physiological function and pathological changes in the body.

### Lactate promotes angiogenesis

**Figure 2:**
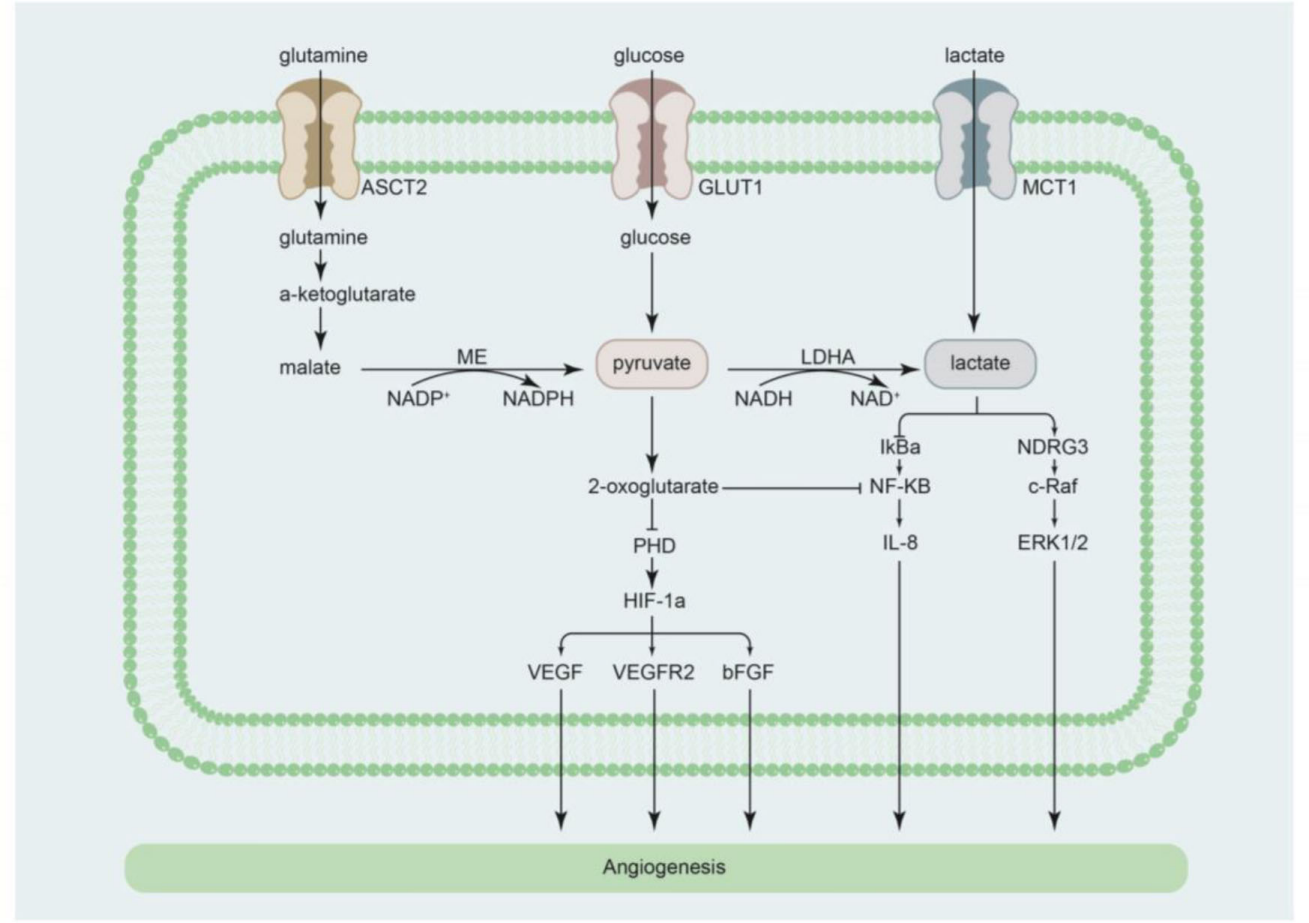
Cellular pathways by which lactate regulates angiogenesis. *ASCT2* alanine serine cysteine transporter 2, *bFGF* basic fibroblast growth factor, *GLUT1* glucose transporter 1, *HIF-1α* hypoxia-inducible factor-1α, *IL-8* interleukin-8, *IκBα* nuclear factor κB inhibitor-alpha, *LDHA* lactate dehydrogenase A, *MCT1* monocarboxylate transporter 1, *ME* malic enzyme, *NDRG3* N-Myc downstream-regulated gene 3, *NF-κB* nuclear factor κB, *PHD* prolyl-4-hydroxylase, *VEGF* vascular endothelial growth factor, *VEGFR2* vascular endothelial growth factor receptor 2.

The promotion of angiogenesis to ameliorate ischemia and hypoxia is beneficial for maintaining cardiovascular function in myocardial infarction (MI), ischemic cardiomyopathy (61), a compensatory period of myocardial hypertrophy (62), and chronic thromboembolic PAH (63, 64). Recent studies have confirmed that lactate promotes angiogenesis (6, 7, 65–69) (**Figure 2**). In preclinical experiments, exogenous supplementation or endogenous production of lactate promoted angiogenesis in brain tumors. In contrast, angiogenesis was impeded by inhibiting lactate production, by knocking down LDHA (70) or lactate transportation by targeting MCT1 (71–75).

The mechanism by which lactate promotes angiogenesis can be partly explained by its effects on signal regulation. Vegran et al. performed both *in vitro* and *in vivo* experiments and reported that when lactate is transported into endothelial cells, it activates pro-angiogenic NF-κB/IL-8 signaling to promote angiogenesis (74). Previous studies showed that lactate inactivates prolyl-4-hydroxylase (PHD) and stabilizes the activation of hypoxia-inducible factor-1α, subsequently promoting angiogenesis by inducing the expression of vascular endothelial growth factor (VEGF)/VEGF receptor 2 and basic fibroblast growth factor (6, 66, 73, 76–78). Of note, lactate can indirectly promote angiogenesis by inducing macrophage secretion of VEGF-α (79, 80). Furthermore, recent studies have determined that lactate promotes angiogenesis *via* direct binding of N-Myc downstream-regulated gene 3 (NDRG3), an oxygen-regulated protein (5, 81). Lactate prevents NDRG3 from degrading and facilitates hypoxia-induced activation of the c-Raf/ERK pathway, which promotes angiogenesis. Inhibition of lactate production abolishes NDRG3-mediated angiogenesis (5, 81). Therefore, lactate may play a cardioprotective role *via* the promotion of angiogenesis, providing a new direction for the prevention and treatment of cardiovascular diseases presenting with ischemia and hypoxia.

### Effects of lactate on hemodynamics

Tissue metabolites regulating hemodynamics are well-documented (82). Under ischemic or hypoxic conditions, a considerably increased lactate production results in vasodilation with a marked reduction in vascular resistance (8, 60). Lactate also has pH-independent vasodilatory effects in animal coronary arteries (83–85). Nonetheless, Brazitikos et al. confirmed that neutralized lactate mediates acute hypoxia-induced vasodilation in the retina (86), suggesting that the vasodilatory effect may be due to lactate independent of pH change (87). Montoya et al.’s *in vitro* experiment revealed that lactate leads to coronary dilatation *via* the release of endothelial nitric oxide in the isolated perfused rat heart (8). Another *in vivo* experiment, conducted by Omar et al, showed that lactate might cause cGMP-mediated vasodilation in calf pulmonary arteries (88). Additionally, further studies have indicated that lactate promotes vasodilation, which is partly mediated by the activation of K_Ca_ channels in porcine coronary arteries (60).

The effects of lactate on vascular resistance and vasodilation are heterogeneous in different organs. Recent studies have shown that GPR81 agonists induce hypertension in rodents, which can be rescued by GPR81 inactivation (20, 89). The pressor effect has been associated with different effects on vascular resistance, which increases in the kidney but remains unchanged in the heart and hind limb (20, 89). This suggests that lactate may bridge metabolism and hemodynamics to maintain body homeostasis under ischemic or hypoxic conditions.

### Regulation of cardiac electrophysiological activity

Lactate regulates the electrophysiological activity of the myocardium and is associated with arrhythmia (9, 10, 90). A possible explanation for this effect is how the lactate affects ion channel regulation (91). ATP-sensitive potassium (K_ATP_) channels are inward-rectifying potassium channels that are widely distributed in cardiomyocytes, VSMCs, non-vascular smooth muscle cells, and nerve cells (92–94). Under physiological conditions, activation of the K_ATP_ channels plays a myocardial protective role; however, its continuous activation can lead to serious ventricular arrhythmias or even ventricular fibrillation (92–94). Keung et al. reported that intracellular application of lactate activates the K_ATP_ channels in guinea pig myocytes (95). Furthermore, Jin et al. confirmed that intracellular lactate induces the opening of the K_ATP_ channels in a dose-dependent manner in rabbit ventricular myocytes (91).

The fast sodium current (I_Na_) is an essential ion channel on the membrane of the fast reactive myocardium and is an important cause of arrhythmia under pathological conditions (96). Lactate can modify I_Na_ by hyperpolarizing guinea pig ventricular myocytes, which may contribute to the development of ischemic arrhythmia (96, 97). Revealing the effects of lactate on cardiac electrophysiological properties will provide new insights into ischemic arrhythmia.

## Implication of lactate in cardiovascular diseases

**Figure 3:**
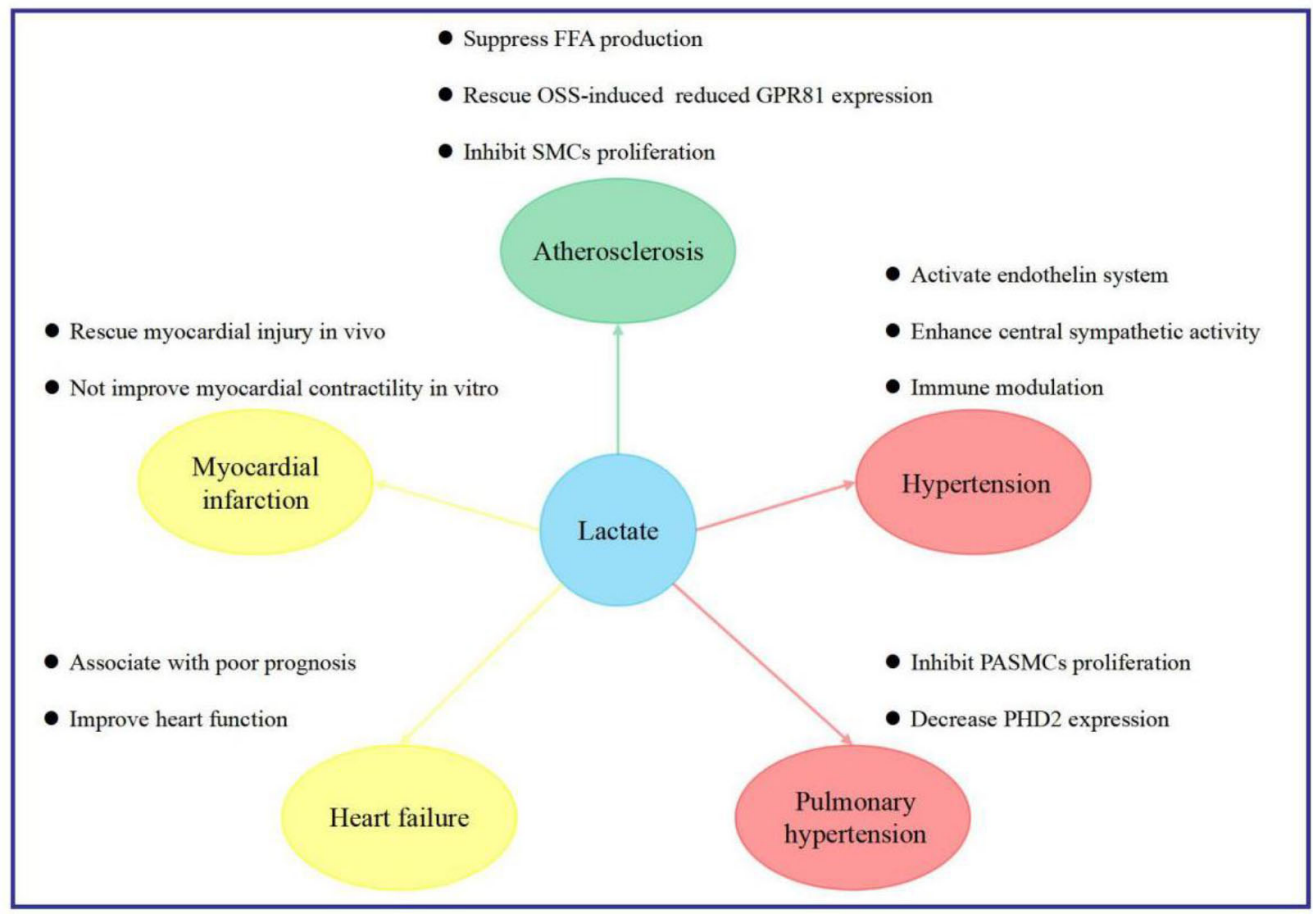
Implications of lactate in cardiovascular diseases. *FFA* free fatty acid, *GPR81* G protein-coupled receptor 81, *OSS* oscillatory shear stress, *PASMCs* pulmonary arterial smooth muscle cells, *PHD2* 2-oxoglutarate-dependent prolyl-4-hydroxylase, *SMCs* smooth muscle cells.

Various cardiovascular diseases are associated with elevated lactate concentrations, which often indicate a poor prognosis. Revealing the potential mechanism of lactate may provide a new understanding and lead to a breakthrough in preventing and treating cardiovascular diseases (**Figure 3**).

### Lactate and atherosclerosis

Wall thickness is a marker of atherosclerotic plaque burden and is strongly associated with clinical events (98–100). The Atherosclerosis Risk in Communities (ARIC) carotid MRI study revealed a strong gradient correlation between lactate and wall thickness (101). In addition, recent studies have shown that lactate may reduce the risk of atherosclerosis by acting *via* a variety of different pathways.

First, plasma free fatty acid (FFA), an important risk factor for atherosclerosis, is mainly derived from triglyceride lipolysis in adipose tissues. Lactate activates GPR81 and suppresses lipolysis through insulin-induced antilipolytic effects in mouse, rat, and human adipocytes, and differentiated 3T3-L1 cells (27, 102, 103). GPR81-selective agonists can suppress lipolysis and FFA production *in vitro* and in mice without side effects (20, 104).

Second, areas of the vasculature affected by oscillatory shear stress (OSS) are more likely to develop into atherosclerotic lesions (105, 106). Physiologically relevant lactate doses can rescue OSS-induced reductions in GPR81 expression, and subsequent GPR81 activation can result in valuable atheroprotective effects in OSS-exposed endothelial cells (107).

Third, SMC proliferation is a key event in atherogenesis. MCT3 mRNA and protein expression are related to atherosclerosis severity. An impairment in lactate transport, arising from MCT3 inhibition, may result in enhanced SMC proliferation and promote of atherosclerosis (108). Lactate exerts an anti-atherosclerotic effect, which may, at least in part, explain the protective effects of moderate exercise against coronary heart disease. This observation suggests that regulation of lactate metabolism may be a potential method for preventing coronary heart disease.

### Lactate and MI

Diabetic patients with acute MI (AMI) have been reported to have higher blood lactate concentrations than non-diabetic patients with AMI (4.54 ± 1.44 vs. 3.19 ± 1.005 mmol/L; *P*<0.05) (109). Blood lactate concentrations in AMI with diabetes are associated with an increased incidence of HF, severe arrhythmias, cardiogenic shock, and a higher mortality rate (109). Yang et al. reported that lactate concentrations, LDH levels, and MCT expression were greater in the ischemic zone than in non-ischemic tissues in a swine MI model (5). Whether the increase in lactate expression plays a protective role or is just a secondary change in AMI remains controversial. Zhang et al. induced AMI in rats and reported that pharmacological preconditioning with lactic acid and hydrogen-rich saline or lactic acid alone could rescue the infarct area, serum myocardial injury markers, and apoptotic index. This was achieved by creating conditions that mimic persistent tissue acidosis and allow for the selective generation of reactive oxygen species (110). However, Aresta et al. identified that increasing tissue lactate concentrations *via* repeated transient lactate exposure did not improve contractile recovery after a prolonged ischemic period in an isolated rat heart model (111). Therefore, more evidence is required to reveal the effects of lactate on AMI, which can be an indicator of prognosis.

### Lactate and HF

It is generally acknowledged that elevated lactate concentrations are common in patients with HF and are related to poor outcomes (112–115). Several known conditions can cause lactate accumulation in patients with HF, including (1) the peripheral tissues lack blood and oxygen supply due to low cardiac output, vasoconstriction, hypoxemia, impairment in tissue perfusion, or inability of tissues to increase oxygen extraction (115–118) (2); adrenergic drive and neurohormonal activation, resulting in higher oxygen demand (113); and (3) diminished lactate clearance ability attributable to abnormal hepatic and renal functions (113, 118). An understanding of the causes of lactate accumulation is conducive to personalized treatment strategies.

Increased blood lactate concentrations (≥2 mmol/L) are correlated with a higher 1-year mortality rate in patients with acute HF (113). According to Kawase et al., elevated lactate levels (>3.2 mmol/L) on admission were related to worse in-hospital mortality (odds ratio, 2.14; 95% confidence interval [CI], 1.10–4.21; *P*=0.03) in patients with acute decompensated HF, either with or without acute coronary syndrome (119) This suggests that high lactate levels could also aid in stratifying the initial risk of early mortality. Gjesdal et al. reached a similar conclusion, reporting that the 30-day mortality rate was higher in MI patients complicated by HF who had a lactate level of ≥2.5 mmol/L than in other patients (112). All of the above mentioned results are based on studies that conducted a single lactate measurement. Biegus et al. investigated how persistent hyperlactatemia affects patients with HF (120) and examined 222 patients with elevated lactate levels. They observed that patients with persistent hyperlactatemia, defined as hyperlactatemia both on admission and after 24 h of hospitalization, had a higher rate of adverse events (e.g., HF worsening) than patients with transient hyperlactatemia. Additionally, persistent hyperlactatemia was an independent predictor of 1-year mortality (hazard ratio [HR], 2.5; 95% CI, 1.5–4.3; *P*<0.001) (120). In addition to the simple increase in lactate levels, Biegus et al. also observed that hyperlactatemia combined with intracellular iron deficiency significantly increased mortality compared to the control group (HR, 5.6; 95% CI, 2.2–14; *P*=0.0003) (114, 121). While hyperlactatemia is associated with a poor prognosis in patients with HF, the available evidence indicating that lactate is a risk factor for HF is difficult to prove because lactate is also an important energy source for the myocardium at rest and during stress (43–45). Danielle et al., using metabolomics to quantify blood metabolites from 110 patients, identified that failing hearts consumed more ketones and lactate (48). A pilot randomized controlled clinical trial identified that infusion of half-molar sodium lactate to patients with acute HF increased cardiac output (from 4.05 ± 1.37 L/min to 5.49 ± 1.9 L/min; *P*<0.01) and tricuspid annular plane systolic excursion (from 14.7 ± 5.5 mm to 18.3 ± 7 mm; *P*=0.02) without any detrimental effects on organ function (36). Furthermore, preclinical experiments revealed that in cardiac myocytes of rats with congestive HF, the MCT1 formation was significantly upregulated, and the lactate uptake rate was increased, which might promote myocardial energy metabolism and improve heart function (122, 123). Whether lactate is a protective or risk factor in patients with HF needs to be further verified.

### Lactate contributes to hypertension

Increasing evidence from clinical and preclinical experiments has shown that lactate contributes to the development of hypertension. In a study that included 5,554 participants from the ARIC study who had no diagnosed or subclinical hypertension at baseline, the mean plasma lactate concentrations were 0.8 mmol/L. Compared to the first quartile, the fourth quartile of plasma lactate concentrations was associated with a higher risk of hypertension at a median follow-up of 11.9 years (HR, 1.18; 95% CI, 1.07–1.31), even after adjustment for conventional risk factors (124). Moreover, Lian et al. reported that plasma lactate concentrations were significantly higher in patients with non-dipping hypertension than in those with dipping hypertension, which may contribute to greater targeted organ damage (125). Furthermore, animal experiments revealed that intravenous injection of sodium lactate at concentrations of 0.5 M or 2 M led to a prompt and short-term increase in blood pressure among normotensive Wistar rats and spontaneously hypertensive rats (126).

Lactate may promote an increase in blood pressure *via* the following pathways. First, lactate is a GPR81 agonist that induces hypertension in wild-type rodents *via* the endothelin system. Also, antagonism of the endothelin receptors can block the increase in blood pressure (20, 89). Second, lactate promotes an elevation of systemic arterial blood pressure by increasing central sympathetic activity. Marina et al. showed that brainstem hypoxia triggers lactate and ATP release in spontaneously hypertensive rats, promoting C1 neuron excitation *in vitro* and increasing sympathetic nerve activity and arterial blood pressure *in vivo* (127). Third, immunomodulation is crucial to the development of hypertension and hypertensive organ injury (128–130). Lactate is an important metabolite that contributes to immunomodulation, which may potentially elucidate why lactate promotes the occurrence of hypertension and target organ injury; nonetheless, further studies are required to confirm this (128, 131, 132). Furthermore, lactic acid can reduce extracellular pH *via* protons, leading to the activation of acid-sensing ion channels and reflexively increasing mean arterial pressure (133).

### Lactate promotes PAH

Using ultra-high-performance liquid chromatography coupled with high-resolution mass spectrometry, our team recently determined that serum lactate concentrations were higher in patients with PAH than in healthy controls (134). We also confirmed that the expression of glycolysis-related enzymes and LDH increased in a rat model of monocrotaline-induced PAH (134). Our results are consistent with the findings of other relevant studies, which reported that the Warburg effect is enhanced with increased lactate generation in PAH (135, 136). It is well documented that PASMC hyperproliferation is an important pathological basis of pulmonary vascular remodeling (137–140). Lactate not only promotes the proliferation of induced pluripotent stem cell-derived VSMCs in the human aorta (5) but also encourages PASMC proliferation and pulmonary vascular remodeling (57). Recent studies have reported that pulmonary vascular endothelial cells from patients with PAH have decreased PHD2 expression and that mice with endothelial cell-targeted disruption of the gene for PHD2 (EGLN1) develop obliterated vascular remodeling and complex lesions, similar to patients with PAH (141). Additionally, lactate can inhibit 2-oxoglutarate-dependent PHD, predominantly PHD2 (3, 66). These experiments suggest that lactate may promote the development of PAH *via* PHD2 inhibition. Recently, some researchers have focused on inhibiting the Warburg effect to develop new therapies for PAH (136, 142). However, prior to that, a more careful investigation of the mechanism of the Warburg effect and lactate in relation to the development of PAH is necessary (136).

## Targeting lactate metabolism and signaling

As mentioned above, lactate affects the progression of cardiovascular diseases. Regulation of lactate production or signal transduction is a promising approach for cardiovascular disease therapeutics. Three pathways can directly influence lactate metabolism and signal transduction.

First, targeting LDH enzymes can directly affect lactate production. A variety of LDH-targeting compounds have been validated in preclinical models of cancer (19). Among these compounds, AT-101 is a nonselective LDH inhibitor, while galloflaavin, FX-11, and N-hydroxyindole-based compounds have been identified to preferentially inhibit LDHA than LDHB (19). Therefore, the efficacy of LDH inhibitors will depend on the expression of the LDH isoform in tissues and will be context-dependent.

Second, targeting MCTs may have considerable effects on lactate-dependent metabolic symbiosis, which is responsible for intracellular and extracellular lactate homeostasis. Several MCT inhibitors have been identified, such as α-cyano-4-hydroxycinnamate (143), organomercurials, and stilbene disulfonates (144), as well as other MCT inhibitors with higher selectivity, includingAR-C155858 (145), and the AstraZeneca compounds AZD3965 (targets MCT1 and MCT2) (146) 7-aminocarboxycoumarins (targets MCT1 and MCT4), SR13800 (targets MCT1) (147), and AZ93 (targets MCT4) (148). Currently, inhibition of MCT transport has been widely investigated in the field of cancer, and some breakthroughs have been achieved (149). Recent work has also shown that inhibiting lactate export by targeting MCT4 can mitigate isoproterenol-induced hypertrophy in cultured cardiomyocytes and in mice (149).

Third, targeting GPR81 is a potentially effective therapeutic approach, as it plays a signal transduction role by activating GPR81, thus indicating the potential of this receptor in controlling hypertension. Numerous studies have reported that lactate promotes hypertension *via* GPR81 (20, 89). Hence, theoretically, selective high-affinity antagonists of GPR81 have promise as antihypertensive drugs. Furthermore, GPR81 knockout mice ‘do not have any obvious difference in cardiovascular phenotype, indicating that pharmacological blockade of the receptor to antihypertensive might limit any important side effects (20). However, no such blockers are currently available to test the validity of this strategy.

## Conclusions

Lactate metabolism plays an important role in regulating the cardiovascular system. Nonetheless, lactate’s mechanism of action at the molecular level in the cardiovascular system is not fully understood. New evidence indicates that lactate can extend its metabolic function to react with cells, tissues, or organs and that lactate regulates different cellular signaling pathways. Recent findings also suggest an important role for lactate in the heart. Growing both embryonic and induced pluripotent stem cells in lactate-supplemented, glucose-free medium allows for their differentiation into cardiomyocytes (49), indicating their critical role in the differentiation of cardiomyocytes. Isoproterenol-induced hypertrophy could be attenuated by inhibiting lactate export (149), suggesting a possible therapeutic strategy by targeting lactate. These findings not only aid us in obtaining a new understanding of how lactate regulates the cardiovascular system but also encourage us to re-examine the role of the Warburg effect in the cardiovascular system. Further exploration of how lactate regulates the cardiovascular system under physiological and pathological conditions and examining whether targeting lactate metabolism, transport, or signal transduction can be exploited as an effective protective strategy against cardiovascular diseases will be meaningful.

## Data availability statement

The original contributions presented in the study are included in the article/supplementary material. Further inquiries can be directed to the corresponding author.

## Author contributions

PW and FL conceived the idea. PW wrote the manuscript. TZ, YH and PW collected and read the literature and revised the article. FL and ZF read through and corrected the manuscript. All authors contributed to the article and approved the submitted version.
